# Targeting Tumorigenic Coactivators in the PI3K/AKT Signaling Pathway: A Novel Approach for Cancer Treatment

**DOI:** 10.1002/cam4.71304

**Published:** 2025-10-29

**Authors:** Md. Anwarul Haque, Thanasis Poullikkas, F. M. Al‐Amin Kaisar, Alam Khan, Shariful Haque, Murshida Mollik, Mst. Jannatul Mowa, Mst. Hajera Khatun, Al Mamun, Mst. Boby Aktar Bithy

**Affiliations:** ^1^ Department of Pharmacy, Faculty of Science University of Rajshahi Rajshahi Bangladesh; ^2^ Department of Experimental Pathology, Faculty of Medicine University of Tsukuba Tsukuba Japan; ^3^ Department of Biochemistry and Systems Biomedicine Juntendo University Graduate School of Medicine Tokyo Japan; ^4^ Nakatani Biomedical Spatialomics Hub Juntendo University Graduate School of Medicine Tokyo Japan; ^5^ Department of Pharmacy Pabna University of Science and Technology Pabna Bangladesh; ^6^ Department of Pharmacy Manarat International University Dhaka Bangladesh; ^7^ Department of Pharmacy, School of Science and Technology Varendra University Rajshahi Bangladesh

**Keywords:** PI3K/AKT signaling, SALL4, TCL1B, TGF‐β, TMEPAI

## Abstract

**Objective:**

This study aims to explore the persistent activation of the PI3K/AKT signaling pathway in various cancers, with a focus on upstream coactivators that drive tumor growth and contribute to therapeutic resistance.

**Methods:**

An integrated overview of four disparate tumorigenic coactivators of PI3K/AKT signaling, namely, TMEPAI (a transmembrane adaptor protein), SALL4 (a zinc‐finger transcription factor), TCL1B (an oncoprotein coactivator), and TGF‐β (a cytokine ligand) was identified and analyzed through a comprehensive literature review. Their mechanistic insights, signaling interactions, and therapeutic opportunities were also summarized.

**Results:**

The study outcomes demonstrate that each of these coactivators contributes to PI3K/AKT pathway hyperactivation and cancer progression through distinct mechanisms, such as the downregulation of negative regulators or direct enhancement of AKT activation. Emerging therapeutic approaches targeting these coactivators through gene silencing, small‐molecule inhibitors, and peptide‐based interventions were also outlined, along with associated challenges such as drug specificity, toxicity, and resistance.

**Conclusion:**

By synthesizing evidence across these diverse molecules, this review highlights the convergent impact of multiple molecular classes on the PI3K/AKT pathway and outlines future perspectives for leveraging these insights in targeted cancer therapies.

## 1 Introduction

Cancer is a complex multifactorial disease that affects a significant number of individuals worldwide [1]. Despite notable strides in cancer research and treatment, cancer incidence continues to rise, and it remains one of the leading causes of mortality globally [2]. Developing effective cancer therapies is a major challenge in cancer research, and efforts to discover new therapeutic targets are going on [3]. One promising target is the PI3K/AKT signaling pathway, which plays a crucial role in regulating cellular processes such as proliferation, survival, and metabolism.

The PI3K/AKT signaling pathway begins with the activation of phosphoinositide 3‐kinase (PI3K), which converts phosphatidylinositol‐4,5‐bisphosphate (PIP2) to phosphatidylinositol‐3,4,5‐trisphosphate (PIP3). PIP3 then recruits AKT to the cell membrane, where AKT is phosphorylated and activated by phosphoinositide‐dependent kinase 1 (PDK1) and the mammalian target of rapamycin complex 2 (mTORC2) [4]. Activated AKT promotes cell growth and survival by phosphorylating and activating downstream effectors including mTORC1, glycogen synthase kinase 3β (GSK3β), and FOXO transcription factors [5].

Dysregulation of the PI3K/AKT signaling pathway is often observed in different types of cancer, including breast, ovarian, prostate, colorectal cancer, and blood cancer [6]. This dysregulation may result from multiple mechanisms, such as gene mutations affecting PI3K and AKT, amplification of the AKT gene, loss or mutation of negative regulators such as PTEN and PHLPP1, and altered activity of upstream coactivators [7, 8]. Activation of the PI3K/AKT pathway promotes tumor growth and survival, making it a desirable target for cancer treatment [5].

The PI3K/AKT pathway has been a focal point in cancer research for many years, with significant efforts directed towards developing inhibitors that target this pathway [9, 10]. Although several PI3K/AKT inhibitors have been developed and tested in preclinical and clinical studies, their utility has been hampered by toxicity and a lack of selectivity for cancer cells [11]. Since PI3K/AKT signaling is essential for normal cells, targeting these fundamental molecules may cause severe side effects. Therefore, developing effective anticancer therapies with reduced adverse effects necessitate targeting the driver molecules responsible for the hyperactivation of PI3K/AKT signaling in cancer cells.

In recent years, targeting coactivators of the PI3K/AKT pathway has emerged as a potential strategy for cancer treatment [5]. Coactivators play a crucial role in regulating the activity of the PI3K/AKT pathway by facilitating the recruitment and activation of downstream effectors [12]. In theory, inhibiting such coactivators could provide a more cancer‐selective strategy for dampening PI3K/AKT signaling. Several key coactivators of PI3K/AKT have been identified, notably TMEPAI, TGF‐β, SALL4, and TCL1B, each of which can directly or indirectly enhance this pathway. These four molecules represent very different molecular classes: TMEPAI is a transmembrane protein, TGF‐β is a secreted ligand, SALL4 is a transcription factor, and TCL1B is an intracellular oncoprotein coactivator (Table 1). For the purposes of this review, we refer to all of them broadly as coactivators of PI3K/AKT signaling, acknowledging their distinct mechanisms of action. Despite their diversity, each contributes to sustained PI3K/AKT pathway activation in cancer.

**TABLE 1:** A summary of the four key PI3K/AKT coactivators, including their molecular class, mechanism of pathway activation, negative regulators affected, and associated cancer types.

Activator	Molecular class	Mechanism of PI3K/AKT activation	Negative regulators affected	Associated cancer types
TMEPAI (PMEPA1)	Transmembrane adaptor protein	Induces degradation of PTEN and PHLPP1 via recruitment of NEDD4 E3 ubiquitin ligase, relieving feedback inhibition on AKT (promotes full AKT activation)	PTEN, PHLPP1 (protein levels reduced)	Breast (especially triple‐negative), lung, colorectal, ovarian, renal, prostate cancers
SALL4	Zinc‐finger transcription factor	Suppresses expression of PTEN and elevates Bmi‐1, leading to downregulation of PHLPP1; overall effect is removal of AKT inhibitory checks and enhanced AKT signaling	PTEN, PHLPP1 (expression suppressed)	Leukemias (AML), hepatocellular carcinoma, colorectal, breast, endometrial, lung, and brain (glioma) cancers
TCL1B	Oncoprotein coactivator (Akt‐binding protein)	Binds directly to the PH domain of AKT, promoting AKT membrane localization and facilitating AKT phosphorylation by PDK1 (acts as a co‐activator of AKT kinase activity)	*(None directly)* (bypasses PTEN/PHLPP1; amplifies AKT activation)	T‐cell and B‐cell malignancies (T‐PLL, CLL, diffuse large B‐cell lymphoma, other lymphomas)
TGF‐β	Cytokine (transforming growth factor β ligand)	Activates PI3K via a noncanonical pathway: TGF‐β receptor signaling recruits TRAF6, which polyubiquitinates PI3K's p85α subunit, leading to PI3K activation and downstream AKT phosphorylation	*(None directly)* (activates PI3K/AKT independent of PTEN)	Advanced solid tumors (prominent in late‐stage and metastatic cancers of various organs, e.g., breast, lung, pancreas)

This review aims to explore the potential role of each coactivator in PI3K/AKT activation and cancer development. Furthermore, this review will also discuss the potential benefits of targeting these coactivators in PI3K/AKT‐mediated cancers, along with the associated challenges and opportunities.

## 2 PI3K/AKT Signal Activation and Cancer

In 1977, AKT was first identified and named by Staal and colleagues [13]. Later, in 1991, AKT was established as a widely expressed protein kinase by three research groups, including Cantley's team, which isolated an enzyme called PI3K. Richard Roth and his collaborators reported the involvement of insulin in AKT activation. Subsequently, many researchers found that PI3K generates phospholipids which are essential components for AKT activation [14].

**FIGURE 1:**
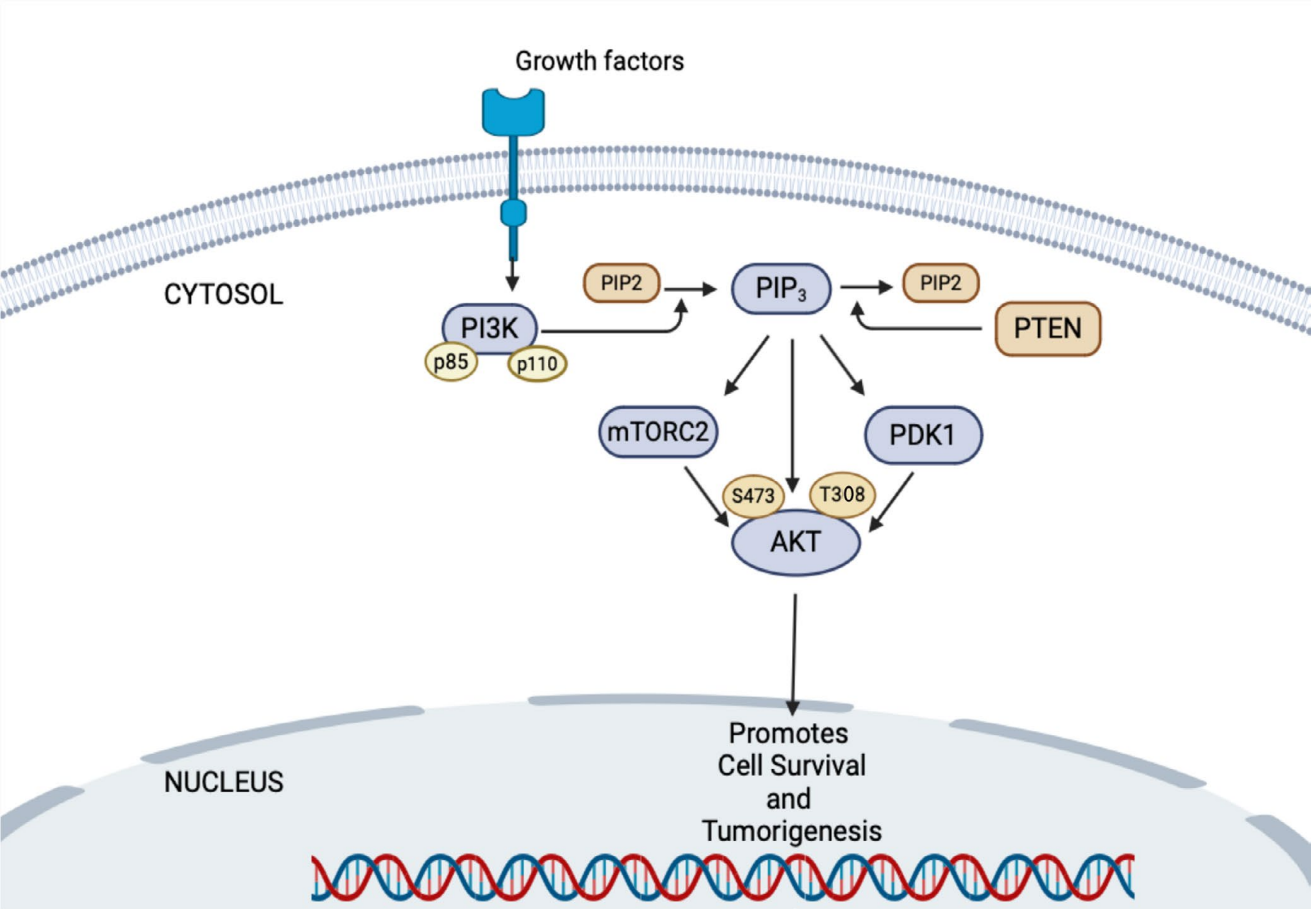
Schematic diagram of the PI3K/AKT signaling activation. Activated RTKs by growth factors recruit PI3K, which facilitates the conversion of PIP2 to PIP3. PIP3 makes conformational changes of PDK1, which in turn activates AKT by phosphorylating the T308 site. The mTORC2 complex, through an unknown mechanism, phosphorylates the hydrophobic motif (Ser473) of AKT and fully activates AKT signaling.

To activate PI3K signaling, receptor tyrosine kinases (RTKs) and other cell surface receptors engage with extracellular ligands [15], and recruit PI3K to plasma membrane‐anchored receptors for the production of phosphatidylinositol (3,4,5) trisphosphate [PI(3,4,5)P3]. PI(3,4,5)P3 then binds with multiple downstream effectors including phosphoinositide‐dependent kinase (PDK) and AKT, recruiting them to the membrane. At this point, PDK phosphorylates AKT at the activation loop (Thr308) [16], whereas the mammalian target of rapamycin complex 2 (mTORC2) actively localizes at the plasma membrane and endosomal vesicles to further phosphorylate AKT at the hydrophobic motif (Ser473) [17], thereby hyperactivating AKT (Figure 1).

Hyperactivation of the AKT signaling pathway is a hallmark of cancer and is associated with uncontrolled cell growth and division. Many types of cancer have been linked to hyperactivated AKT, including breast, ovarian, lung, prostate, brain, pancreatic, and blood cancer. Estimating the exact percentage of different cancers caused by hyperactivated AKT is challenging, as the role of this pathway can vary depending on the specific type and stage of cancer. However, research has shown that hyperactivation of AKT is a common feature of many cancer types and can contribute to cancer progression and resistance to therapy. For example, in breast cancer, up to 70% of cases show hyperactivated AKT. In prostate cancer, approximately 40% of early‐stage and 70%–100% of advanced‐stage cases exhibit hyperactivation. Serous ovarian cancer sees approximately 45% of high‐grade cases with hyperactivated AKT. Non‐small‐cell lung cancer has around 50%–73% of cases with this feature, while brain cancer, specifically glioblastoma multiforme (GBM), shows around 88% [18, 19, 20, 21]. In pancreatic cancer, more than 40% of cases have hyperactivated AKT. Hyperactivation of AKT has been linked to increased tumor growth, metastasis, and resistance to chemotherapy. While the exact percentage of different cancers due to hyperactivated AKT is not clear, it is evident that this pathway plays a significant role in cancer development and progression, making it a promising target for cancer therapy.

## 3 Clinical and Preclinical Agents Targeting PI3/AKT Signaling

A diverse array of small‐molecule inhibitors and monoclonal antibodies has been developed to target various components of the PI3K/AKT pathway. Preclinical studies have demonstrated the efficacy of these agents in inhibiting tumor growth, inducing apoptosis, and overcoming resistance to standard therapies in various cancer models. For example, PI3K inhibitors such as idelalisib and alpelisib have shown promising activity in preclinical models of hematologic malignancies and solid tumors by selectively targeting PI3K isoforms [22, 23]. Similarly, AKT inhibitors such as ipatasertib and capivasertib have demonstrated potent antitumor effects by inhibiting AKT phosphorylation and downstream signaling.

Numerous clinical trials have been conducted to evaluate the safety and efficacy of PI3K/AKT inhibitors in cancer patients. These trials have investigated a wide range of PI3K/AKT inhibitors as monotherapy or in combination with other anticancer agents across different cancer types, including breast cancer, colorectal cancer, lung cancer, and melanoma. Notable examples include the phase III SOLAR‐1 trial [24], which demonstrated improved progression‐free survival with the addition of alpelisib to fulvestrant in PIK3CA‐mutant [22] hormone receptor‐positive breast cancer patients, and the ongoing IPATunity130 [25] trial evaluating ipatasertib in combination with paclitaxel for advanced triple‐negative breast cancer.

Clinical trials evaluating agents targeting the PI3K/AKT pathway in cancer therapy have yielded a range of outcomes, showcasing both promise and challenges in this field. Buparlisib (BKM120), an oral pan‐class I PI3K inhibitor, has shown potential in phase II trials for advanced breast cancer [26], glioblastoma [27] and head and neck squamous cell carcinoma (HNSCC) [28]. However, phase III trials like BELLE‐2 and BELLE‐3 [29] in breast cancer failed to meet primary endpoints, underscoring the complexities of targeting this pathway effectively. The utilization of increasingly specific PI3K inhibitors, like α‐specific PI3K inhibitors, is justified to enhance safety and efficacy in this context. Due to the toxicity associated with this combination, no additional investigations are being pursued. Additionally, Voxtalisib (XL765), a dual PI3K/mTOR inhibitor, has demonstrated manageable toxicity profiles and early signs of efficacy in phase I trials across various solid tumors [30], high‐grade gliomas [31], and in phase II for lymphomas [32]. Ongoing phase II trials aim to further assess its effectiveness, either as monotherapy or in combination with other agents, in breast cancer, melanoma, and glioblastoma. Gedatolisib (PF‐05212384), another dual PI3K/mTOR inhibitor, has shown promising preclinical activity and safety in phase I trials for solid tumors such as breast cancer [33], ovarian cancer [34], and non‐small cell lung cancer (NSCLC) [35]. Phase Ib trials are underway to evaluate its efficacy, particularly in PIK3CA‐mutant breast cancer [33] and NSCLC [35]. Moreover, Miransertib (ARQ 092), a selective allosteric AKT inhibitor, has demonstrated acceptable safety and initial signs of efficacy in phase I trials for various solid tumors [36], lymphoma [37] and glioma [38]. Ongoing phase Ib trials are assessing its potential in PIK3CA‐mutant solid tumors [39], including ovarian and endometrial cancer [39]. Lastly, Capivasertib (AZD5363), an oral AKT inhibitor, has shown promise in phase I trials for PIK3CA‐mutant breast cancer and other solid tumors [40], with ongoing phase II trials like FAKTION (Capivasertib) [41] in breast cancer. These trials aim to further elucidate its efficacy in combination with standard therapies.

In conclusion, the diverse array of clinical agents targeting the PI3K/AKT pathway in cancer therapy highlights ongoing efforts to translate preclinical findings into clinically meaningful outcomes. Continued research and clinical trials are crucial to optimizing the use of these inhibitors and identifying predictive biomarkers for therapeutic response, ultimately maximizing benefit for cancer patients.

## 4 PI3K/AKT, PTEN, and PHLPP1

**FIGURE 2:**
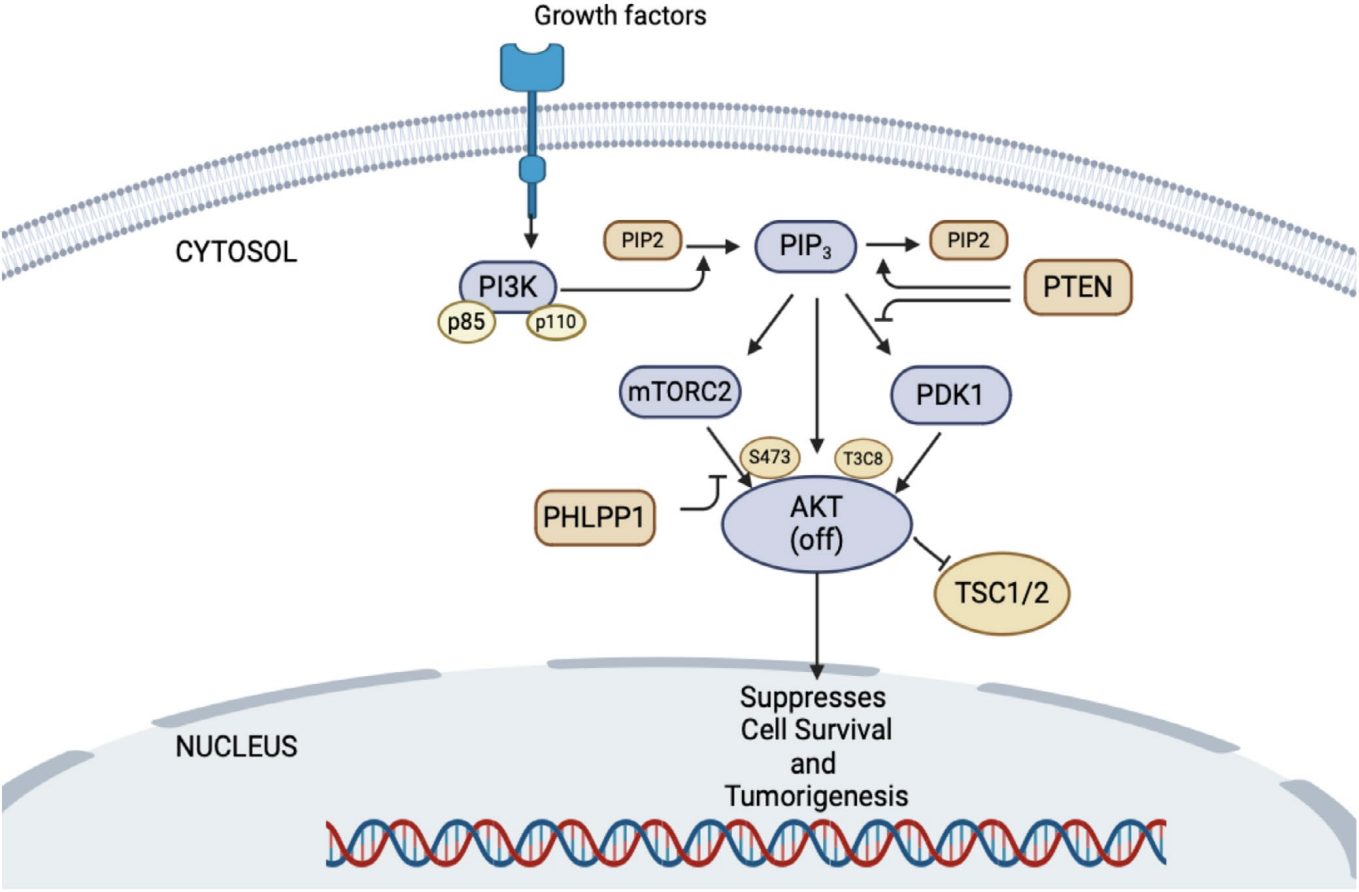
Schematic illustration of inhibition of PI3K/AKT signaling by PTEN and PHLPP1. PTEN, a phosphatase, enhances the transformation of PIP3 to PIP2, and insufficient PIP3 makes it impossible to change the conformation of PDK1 and AKT, which inhibits phosphorylation at the T308 site of AKT. On the other hand, PHLPP1 directly dephosphorylates the Ser473 site of AKT and thus inactivates the PI3K/AKT pathway. Inactivated PI3K/AKT signaling makes cells unable to achieve uncontrolled cell growth and tumorigenesis.

As discussed earlier, the PI3K/AKT pathway cascade is often disrupted and over‐activated in various types of cancer, leading to the development of tumors and resistance to drugs. Targeting this pathway could be an effective cancer therapy [10]. However, developing targeted therapies based on AKT has proven challenging due to its complex cascades, essential role in normal cellular function, and potential side effects of therapies closely connected with signaling suppressors. Two important phosphatases that hinder AKT activation are extensively studied: PTEN and PHLPP1. PTEN is a well‐known tumor suppressor that inhibits AKT activation [42], particularly on Thr308, by counteracting the function of PI3K (Figure 2). On the other hand, PHLPP1 dephosphorylates AKT (Figure 2) at Ser473 [43, 44]. In malignant cells, PI3K/AKT signaling is often activated through various mechanism, such as hyperactivation of RTKs, activating mutations of PI3K, or loss/mutation of PTEN and/or PHLPP1. Additionally, overexpression of driver molecules such as TMEPAI, TGF‐β, SALL4, and TCL1B can contribute to sustained activation of PI3K/AKT signaling. Therefore, identifying the molecules responsible for continuous activation of PI3K/AKT signaling and developing targeted therapies against them represents a promising avenue of research. Such efforts could potentially mitigate PI3K/AKT‐dependent cascades in various cancers, offering new strategies for therapeutic intervention in the future.

## 5 Transmembrane Prostate Androgen‐Induced Protein (TMEPAI)

**FIGURE 3:**
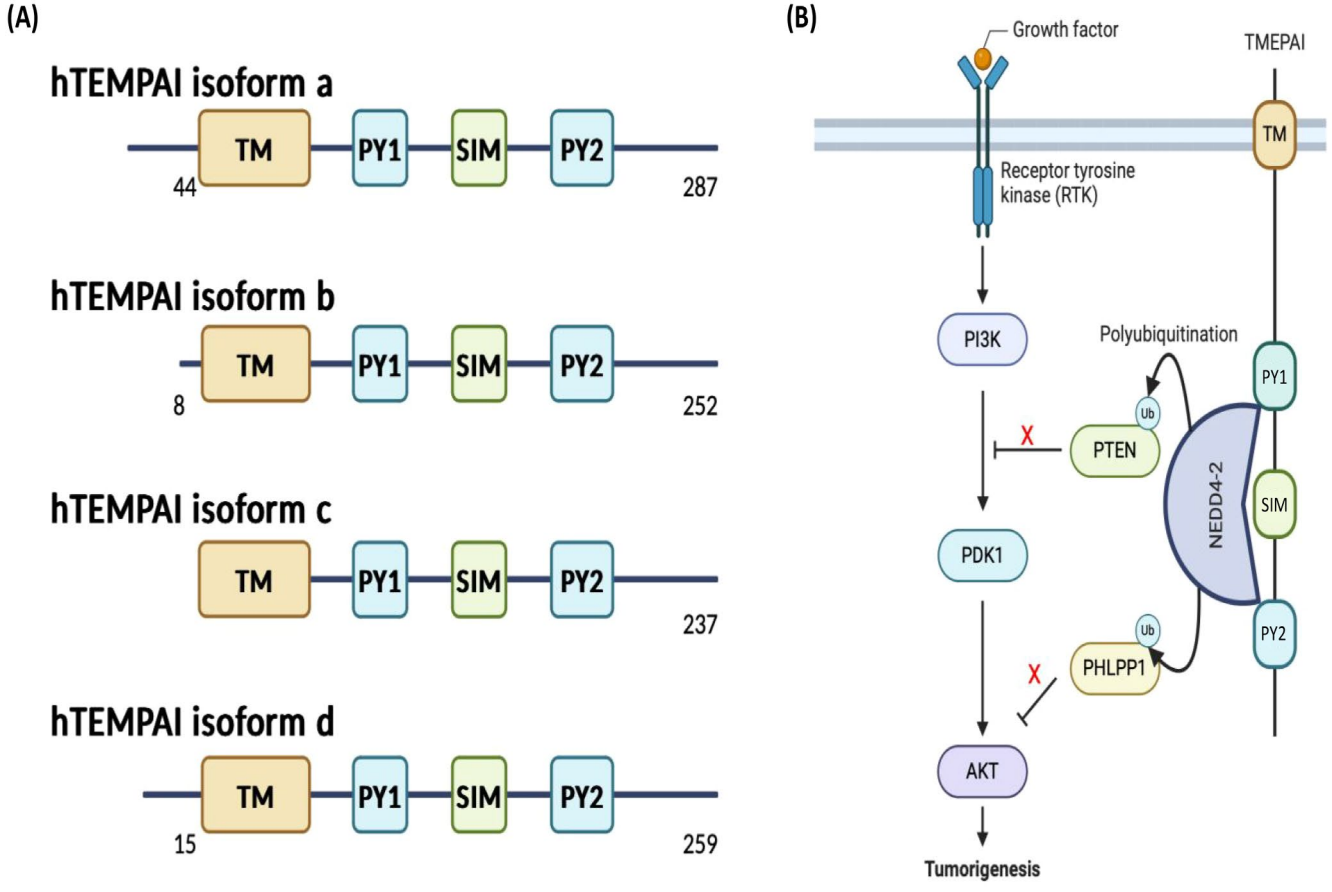
Isoforms of TMEPAI and its role in activating the PI3K/AKT pathway. (A) To date, there are four isoforms of human TMEPAI (a, b, c, and d) with distinct N termini that have been identified. All isoforms have two PY motifs (PY) and one Smad interaction motif (SIM) in their intracellular region. (B) TMEPAI expression is increased in cancer cells, and the two PY motifs of TMEPAI interact with NEDD4‐2 E3 ligase, potentiating PTEN and PHLPP1 degradation, and leading to full activation of AKT signaling, which promotes tumorigenic activities.

The protein TMEPAI is a type‐Ib transmembrane protein that was first identified as an androgen‐induced oncoprotein [45]. It is also known as prostate transmembrane protein, androgen‐induced 1 (PMEPA1) or solid tumor‐associated gene 1 (STAG1). It has a short extracellular domain, followed by a transmembrane domain and a long intracellular domain that contains a Smad interaction motif (SIM) and two PPxY (PY) motifs [46]. Four isoforms of human TMEPAI protein have been identified, all sharing a common intracellular structure with a SIM between two PY motifs [46, 47, 48, 49] (Figure 3A). TMEPAI is highly and constitutively expressed in many cancers including breast, lung, colorectal, ovary, and renal cancers [50, 51, 52, 53, 54, 55]. Its transcriptional induction is regulated by transforming growth factor‐β (TGF‐β) in conjunction with other oncogenic signaling pathways [56, 57, 58]. Previous studies have shown that depletion of TMEPAI reduces tumorigenic activities in various cancer cells by modulating intracellular signaling pathways [50, 51, 54]. Singha et al. [52] demonstrated that TMEPAI downregulates PTEN to promote PI3K/AKT signaling, while Haque and his colleagues [11] found that TMEPAI activates PI3K/AKT signaling by facilitating NEDD4 mediated proteasomal degradation of PHLPP1, thereby fully activating PI3K/AKT signaling (Figure 3B). Therefore, TGF‐β‐induced TMEPAI is considered a crucial oncogenic coactivator of AKT and a promising target for developing molecular targeted therapy aimed at managing and treating PI3K/AKT signaling‐driven cancers.

## 6 Sal‐Like Protein 4 (SALL4)

SALL4 is a zinc‐finger transcription factor essential for maintaining the self‐renewal and pluripotency of embryonic stem cells [59]. SALL4 expression normally decreases after embryonic development and is largely absent in most adult tissues [60]. However, aberrant re‐expression of SALL4 has been observed in a wide range of cancers. High levels of SALL4 have been reported in acute myeloid leukemia (AML) [61, 62], liver cancer [63], colon cancer [64], breast cancer [65], endometrial cancer [66], lung cancer [67], and gliomas [68]. In these contexts, SALL4 plays crucial roles in promoting cancer cell survival, proliferation, metastasis, and therapy resistance. Clinically, SALL4 overexpression is associated with aggressive disease and poor prognosis in several cancers. Consequently, SALL4 has been proposed as a potential biomarker for early cancer diagnosis and as a therapeutic target for treatment [69, 70, 71].

**FIGURE 4:**
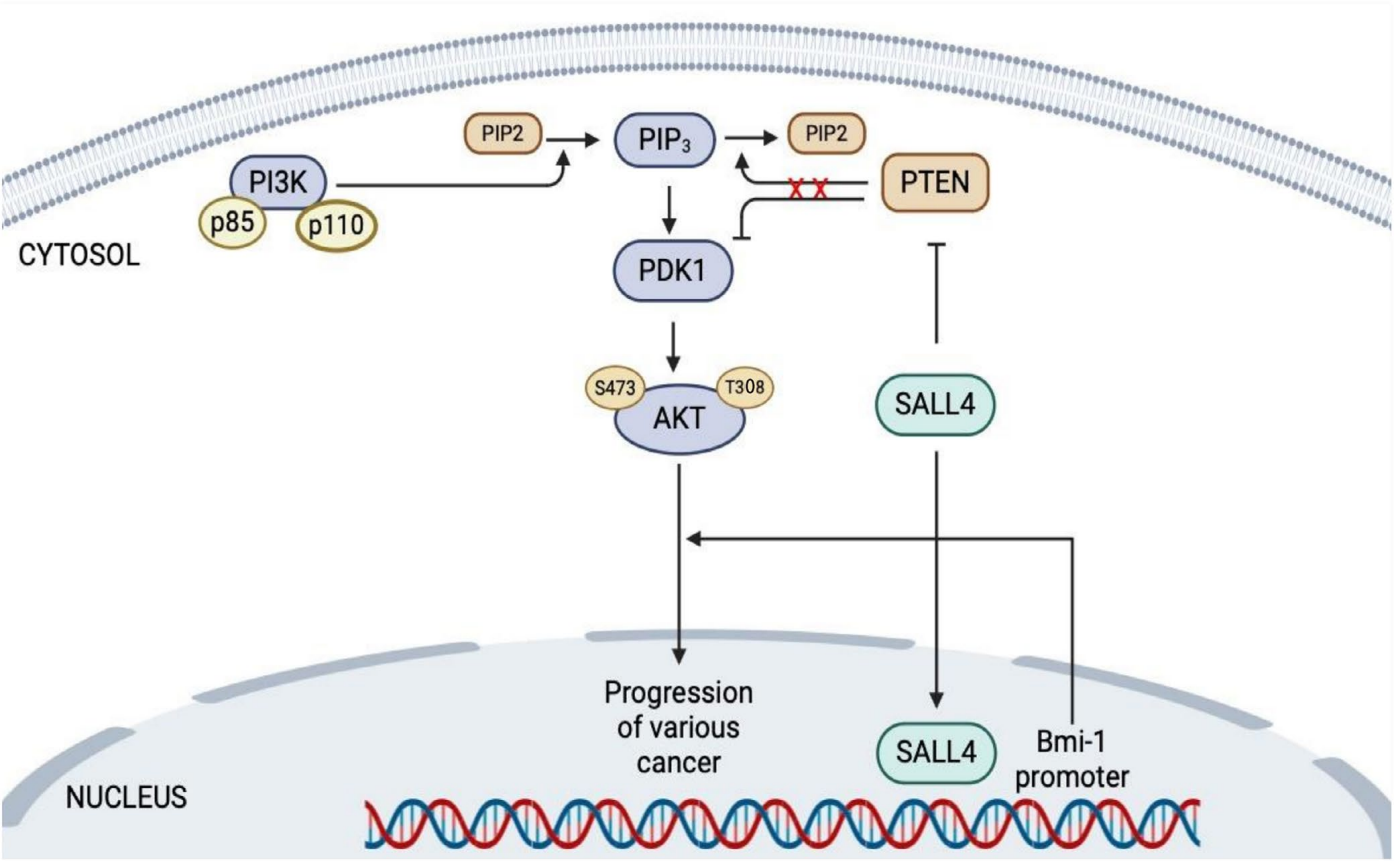
SAll4 mediated activation of PI3K/AKT pathway. SALL4 suppresses PTEN expression and promotes AKT activation. SALL4 directly binds to Bmi‐1, which activates AKT signaling by downregulating PHLPP1 and PTEN.

Recent studies have shed light on how SALL4 contributes to PI3K/AKT pathway activation in cancer. SALL4 appears to enhance AKT signaling through at least two mechanisms. In one mechanism, SALL4 directly upregulates expression of the Bmi‐1 oncogene (by binding to the Bmi‐1 promoter) [72, 73]. Bmi‐1 is part of the Polycomb complex and can activate PI3K/AKT signaling by downregulating the pathway's inhibitors PTEN and PHLPP1. Thus, SALL4‐driven Bmi‐1 expression leads to suppression of PTEN and PHLPP1, relieving their inhibitory effect on AKT (Figure 4). This allows downstream targets of AKT (such as mTOR and GSK3β) to become active, promoting cancer cell growth and survival. In a second, related mechanism, there is an inverse relationship observed between SALL4 and PTEN levels: SALL4 overexpression directly or indirectly represses PTEN transcription [74, 75, 76], resulting in sustained PI3K/AKT activation. Conversely, silencing of SALL4 can increase PTEN expression and reduce AKT activity in cancer cells [70, 77]. Through these routes, SALL4 acts as an upstream coactivator of the PI3K/AKT pathway. Targeting SALL4 and its downstream effects could therefore be a promising strategy to reduce hyperactive AKT signaling in tumors. Given that SALL4 is largely absent in normal adult tissues, therapies against SALL4 might achieve tumor specificity, an aspect further discussed in later sections.

## 7 T Cells Lymphoma/Leukemia 1B (TCL1B)

TCL1B belongs to the TCL1 family of oncoproteins, plays a significant role in the development of leukemia and lymphoma [78, 79]. Various studies have demonstrated that TCL1B can activate the AKT signaling pathway [80, 81] through directly binding to the PH domain of AKT, promoting its phosphorylation by PDK1 [82, 83, 84]. This leads to the activation of downstream targets of the AKT pathway, such as mTOR and GSK3β, which promote cell growth, protein synthesis and cell survival. Inhibiting the AKT pathway sensitizes leukemia and lymphoma cells to chemotherapy and improves overall survival in animal models. Further research is necessary to better understand the mechanisms by which TCL1B regulates the AKT pathway. Targeting the TCL1B‐mediated AKT signaling could potentially be a novel therapeutic approach for the treatment of leukemia and lymphoma.

## 8 Transforming Growth Factor‐β (TGF‐β)

Initially, TGF‐β was discovered to stimulate the growth of normal rat kidney cells when combined with TGF‐α. The TGF‐β family is composed of 33 ligands, including TGF‐βs and pluripotent cytokines [85]. These ligands regulate cell fate and plasticity in both normal tissues and tumors. However, abnormal TGF‐β expression is linked to various life‐threatening diseases, including cancer [86, 87, 88]. Numerous studies have demonstrated the correlation between TGF‐β signaling and cancer. In the early stages of cancer, TGF‐β signaling can trigger apoptosis by inhibiting c‐Myc and increasing DAPK and DAXX protein levels [89]. However, in later stages, TGF‐β acts as a tumor promoter [90]. It is frequently upregulated in the later stages of cancer and is associated with a shorter survival rate, indicating its tumor‐promoting role [91]. Additionally, TGF‐β strongly induces the epithelial to mesenchymal transition (EMT), which promotes cancer invasion and metastasis [92, 93].

**FIGURE 5:**
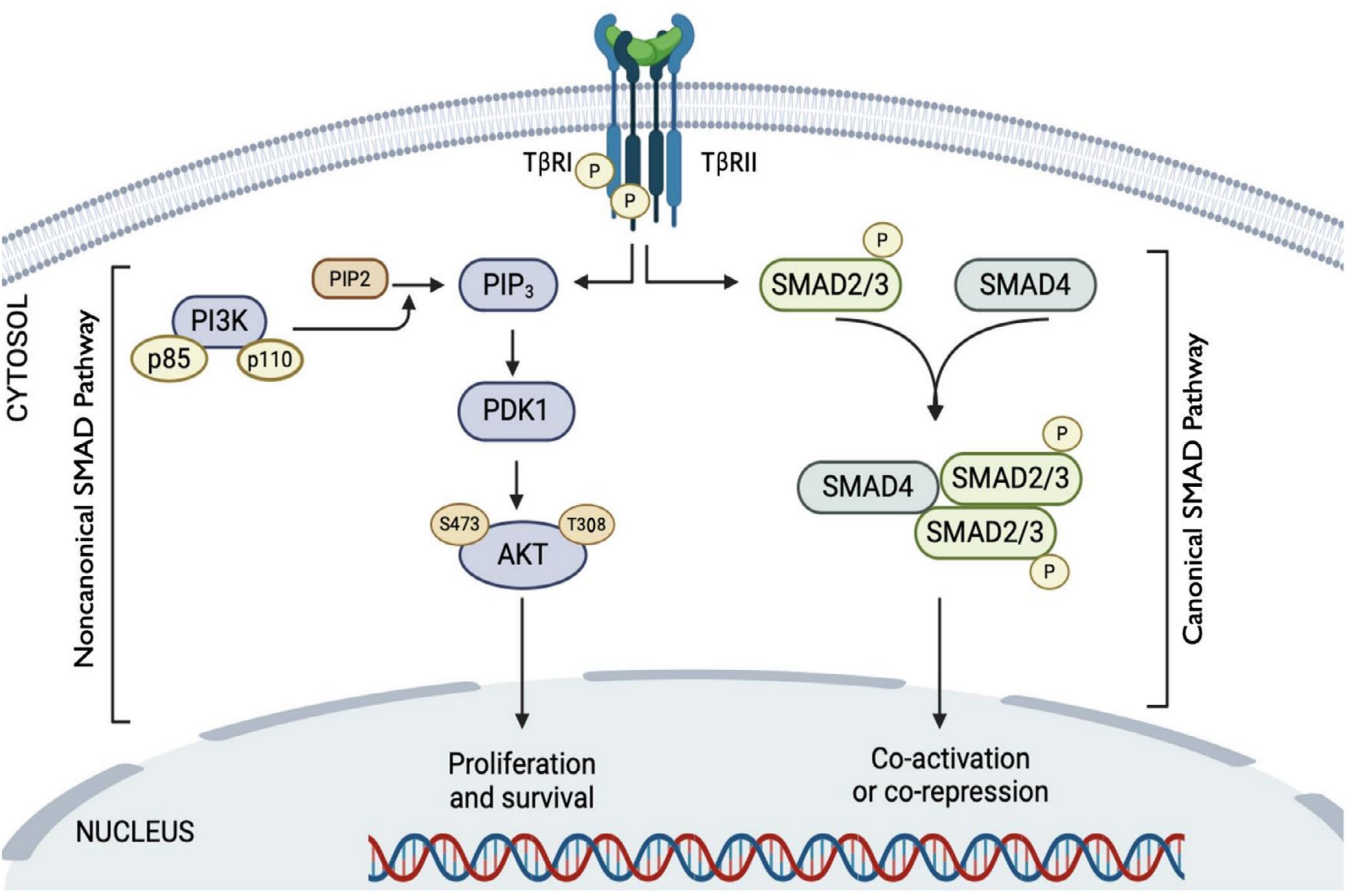
Schematic representation of TGF‐β/Smad signaling. Hetero‐tetrameric form of TGF‐β receptors promotes R‐Smads and Co‐Smad (Smad4) complex formation that enters the nucleus to trigger transcription of various target genes (SMAD pathway), whereas phosphorylated TGF‐β type I receptor (TβRI) activates PI3K, which leads to phosphorylation of AKT.

TGF‐β mediates a range of cellular responses through the canonical SMAD pathway as well as noncanonical pathways such as the PI3K‐AKT pathway. The dimeric form of TGF‐β binds to two TβRI and two TβRII receptors, activating TβRII kinase [94] which phosphorylates TβRI. Phosphorylated TβRI enhances the interaction between receptor‐regulated SMADs (R‐Smads, specifically Smad2 and Smad3) and the common‐mediator SMAD (Co‐Smad, Smad4), constituting the SMAD signaling pathway. The complex then translocates into the nucleus, where it regulates TGF‐β target genes [95] (Figure 5). Inhibitory SMADs, however, suppress TGF‐β signaling [96, 97]. Recent studies have also revealed [98] that TGF‐β activates PI3K in a TRAF6‐dependent manner by polyubiquitinating the PI3K regulatory subunit p85α, resulting in the activation of PI3K and AKT (Figure 5). This represents a noncanonical SMAD pathway. Targeting TGF‐β could be a promising strategy to counteract TGF‐β‐mediated overactivation of the PI3K/AKT signaling pathway in cancer.

## 9 Challenges and Future Perspective

Targeting the upstream coactivators of the PI3K/AKT signaling pathway is an appealing strategy for developing novel cancer therapeutics. By blocking the aberrant activation of PI3K/AKT, it may be possible to inhibit the growth and survival of cancer cells while sparing normal cells. However, several challenges must be addressed to successfully translate this approach.

One primary challenge is determining which PI3K/AKT coactivators are selectively upregulated in cancer cells but not in normal cells. The four coactivators discussed each have different expression patterns in normal physiology. A deeper understanding is needed of the context‐specific regulation of these molecules. For instance, SALL4 is largely silent in adult somatic tissues, whereas TGF‐β is ubiquitous. Defining the context in which each coactivator is pathologically active will help in selecting appropriate therapeutic targets and predicting toxicities. This requires ongoing research into the molecular mechanisms that drive PI3K/AKT hyperactivation in different cancers and into the tissue specificity of each coactivator's expression.

Developing therapies that selectively target these coactivators without affecting other crucial pathways is complex. Many of these molecules engage in multiple cellular functions. Ensuring specificity will be essential to avoid collateral toxicity. For example, a drug that inhibits SALL4 must minimally affect other zinc‐finger proteins or general transcription, and TGF‐β pathway inhibitors must be designed to minimize immune system perturbation. Achieving this selectivity may involve designing inhibitors that exploit unique structural features or using targeted delivery methods. Rigorous preclinical validation of specificity and off‐target profiles will be needed.

Just as cancers can develop resistance to direct PI3K or AKT inhibitors, they might also evade upstream‐targeted therapies. Tumor cells could upregulate compensatory pathways or alternate coactivators if one is inhibited. For instance, blocking SALL4 might lead a cancer cell to rely more on other oncogenic pathways or inhibit TMEPAI's effect on PTEN. For example, targeting miR‐21 and miR‐95 showed a decrease in AKT phosphorylation through PTEN, SNX1, and SGPP1 elevation [99]. Combination therapies will likely be required to address such adaptability. A potential strategy is to combine a coactivator inhibitor with a PI3K/AKT pathway inhibitor or with inhibitors of parallel pathways, such as MAPK or mTOR pathways, thereby reducing the likelihood that the tumor bypasses the blocked node. Future research should also focus on identifying biomarkers of resistance and strategies to counteract them, such as sequential or multi‐target treatment regimens.

Moreover, it will be crucial to develop reliable biomarkers to predict which patients are most likely to benefit from targeting a given PI3K/AKT coactivator. For example, measuring SALL4 levels in tumors could identify patients who might respond to a SALL4‐targeted therapy. Similarly, TCL1B expression or gene rearrangements could guide the use of TCL1B‐targeted strategies in lymphoid malignancies. Biomarker‐driven patient selection is crucial for both clinical trial design and eventual clinical use, ensuring that the therapy is applied to cases where the targeted pathway is indeed driving cancer.

An additional consideration is a potential crosstalk between these coactivators and other signaling networks. The PI3K/AKT coactivators do not operate in isolation; TGF‐β induction of TMEPAI illustrates one such interplay, and TCL1B may cooperate with other pathways like NF‐κB in leukemia. It remains to be elucidated whether inhibiting one coactivator could be compensated by upregulation of another or if simultaneous inhibition yields synergistic effects. Future studies should explore possible interactions among TMEPAI, SALL4, TCL1B, and TGF‐β within the same tumor context. Understanding these relationships could reveal new combination strategies or help anticipate resistance mechanisms. Moreover, exploring tissue‐specific therapeutic windows will be important. Some coactivators offer inherently more selective targets. For instance, SALL4 is largely absent from normal adult tissues, except the germline, suggesting that SALL4‐targeted therapies might spare most normal cells, creating a favorable therapeutic window. In contrast, inhibiting a ubiquitous factor like TGF‐β or a critical developmental pathway carries a greater risk of side effects. Tailoring therapy to exploit such differences will be crucial for safety.

Despite the challenges, early‐stage research has begun to explore therapeutic strategies against the PI3K/AKT coactivators.

### 9.1 TMEPAI

Being a transmembrane protein with E3 ligase adaptor function, TMEPAI can be targeted by reducing its expression or blocking its interaction with binding partners. Gene silencing approaches (siRNA or shRNA) against TMEPAI have been shown to suppress tumor cell proliferation and invasion in vitro. Moreover, small‐molecule screens have identified compounds that downregulate TMEPAI. One such compound, a 2‐(2‐nitrobenzylidene) indolin‐3‐one derivative, was found to inhibit TMEPAI promoter activity, leading to reduced TMEPAI mRNA/protein levels and growth arrest of cancer cells [100]. Overexpression of TMEPAI rescinded the drug's effect, and TMEPAI knockdown sensitized cells to it, indicating on‐target action [99]. Although no TMEPAI‐specific therapy has reached clinical trials yet, these findings demonstrate that TMEPAI is a druggable target and provide a starting point for developing inhibitors to block its tumorigenic function.

### 9.2 SALL4

As an “undruggable” transcription factor, SALL4 has attracted interest for RNA‐based and epigenetic therapies. Antisense oligonucleotides (ASOs) and short interfering RNAs (siRNAs) directed at *SALL4* mRNA can effectively reduce SALL4 expression, resulting in impaired cancer cell growth and induced apoptosis in preclinical models. Given SALL4's absence in most adult tissues, ASO/siRNA approaches are particularly promising. In addition, small molecules that indirectly target SALL4 are being explored. A notable example is the histone deacetylase inhibitor entinostat, which was identified via connectivity mapping as a compound that selectively kills SALL4‐positive cancer cells. In preclinical studies on lung cancer, entinostat treatment downregulated SALL4 through upregulation of miR‐205 and preferentially inhibited the growth of SALL4‐expressing cells [101]. SALL4 knockdown or a peptide that interferes with SALL4 function also reduced tumor cell viability in these models [101]. These results lay the groundwork for future clinical studies evaluating SALL4‐targeted strategies. Other avenues, such as disrupting SALL4's interaction with its protein partners or DNA, should also be investigated.

### 9.3 TCL1B

The primary strategy to counter TCL1B's oncogenic effect is to disrupt its cooperation with AKT. One approach has been to design peptides or small molecules that mimic the AKT‐binding interface of TCL1, thereby competitively inhibiting the TCL1–AKT interaction. For instance, a peptide spanning the βA strand of TCL1 (termed “Akt‐in”) was shown to bind to AKT's PH domain and prevent AKT from associating with the membrane, effectively inhibiting AKT activation in vitro. This peptide demonstrated the feasibility of blocking AKT coactivation and even reduced tumor growth in vivo without significant toxicity [102]. Beyond direct inhibition, an indirect approach to target TCL1B is to destabilize it. TCL1B's stability in cells is partly maintained by chaperones such as HSP70. Inhibiting HSP70 with small molecules like myricetin causes TCL1 proteins to become misfolded and tagged for proteasomal degradation. Gaudio et al. [102] showed that treating leukemia/lymphoma cells with an HSP70 inhibitor led to ubiquitination and loss of TCL1, resulting in impaired tumor growth in xenograft models [103]. While no TCL1B‐specific drug has advanced to clinical trials yet, these proof‐of‐concept studies underscore multiple possible routes: disrupt the TCL1B–AKT binding with peptides or analogs or induce TCL1B clearance via modulators of its stabilizing partners. Any TCL1B‐targeted therapy would likely be applied to TCL1‐positive leukemias/lymphomas, and efforts are ongoing to identify lead compounds for this purpose.

## 10 Conclusion

In conclusion, the diverse upstream coactivators converging on PI3K/AKT signaling underscore the complexity and redundancy of this oncogenic pathway. By integrating insights from transmembrane adaptor proteins, like TMEPAI, transcriptional regulators such as SALL4, oncoprotein coactivators similar to TCL1B, and cytokine signals akin to TGF‐β, we gain a more holistic understanding of how PI3K/AKT is hyperactivated in cancer. Targeting these coactivators represents a novel and complementary approach to conventional PI3K or AKT inhibition. This strategy holds the appeal of potentially greater cancer specificity. For instance, focusing on a cancer‐specific coactivator may avoid some systemic side effects of direct pathway inhibition. However, it also demands careful consideration of each target's biology, particularly their normal physiological roles, to avoid undesired consequences. Moving forward, continued research is needed to unravel the context‐specific roles of these coactivators, to discover additional regulatory players, and to refine therapeutic agents against them. If successful, such efforts could expand the arsenal of targeted therapies for PI3K/AKT‐driven malignancies. Rather than a one‐size‐fits‐all approach, a personalized strategy may emerge, wherein the particular PI3K/AKT coactivator profile of a patient's tumor guides the choice of therapy. Ultimately, integrating upstream coactivator inhibition with existing treatments could pave the way for more effective and tailored interventions, improving outcomes for patients with cancers linked to the PI3K/AKT pathway.

## Author Contributions

**Md. Anwarul Haque:** conceptualization, designed the study and implemented the search strategy, formal analysis (equal), Software (equal), data curation (equal), led manuscript development and wrote the original draft (equal), review and editing (equal), supervision and project administration. **Thanasis Poullikkas:** formal analysis (equal), Software (equal), data curation (equal), led manuscript development and wrote the original draft (equal), writing – review and editing (equal). **F. M. Al‐Amin Kaisar**, **Shariful Haque**, **Murshida Mollik**, and **Alam Khan:** formal analysis (supporting), data curation (supporting), Software (supporting), original draft (supporting), review and editing (supporting). **Mst. Jannatul Mowa**, **Mst. Hajera Khatun**, **Al Mamun**, and **Mst. Boby Aktar Bithy:** formal analysis (supporting), data curation (supporting), Software (supporting), review and editing (supporting).

## Conflicts of Interest

The authors declare no conflicts of interest.

## Data Availability

Research data is not shared.
